# The Treatment of Hydrocele by Injections with the Hypodermic Syringe, without Evacuation of the Sac

**Published:** 1883-02

**Authors:** T. M. McIntosh

**Affiliations:** Thomasville, Ga.


### THE TREATMENT OF HYDROCELE BY INJECTIONS WITH THE HYPODERMIC SYRINGE, WITHOUT EVACUATION OF THE SAC.

BY T. M. McINTOSH, M.D., THOMASVILLE, GA.

"Acts are called good or bad, according as they are well or ill adjusted to ends."—*Herbert Spencer.*

I have recently been interested in reading articles upon the subject of hydrocele, because I wished to compare the methods in them advocated, with one which I have used in two cases, which is so much more simple, entirely painless and equally efficient as any method yet known, that I am led to describe it.

In all these readings I find that no one method has been universally used, but departures, more or less wide, from the time honored one of evacuation with trocar and canula and injections with tincture of iodine, have been made.

Volkman has even resorted to the formidable and complicated operation (with use of complete Listerism) of dividing the sac by an incision, beginning near the external abdominal ring and extending downward to the most dependent portion of the sac; and sewing the divided edges of sac and scrotol tissue together—thus exposing the testicle and leaving a linear incision to heal by granulation. The objections to this procedure are obvious and the impracticability of its use by the general practitioner is plain.

The old treatment by trocar evacuation and injecting iodine tincture is generally excessively painful and is not always successful, even after a repetition of the operation.

In the *Medical Record* of July 15th, 1882, is a paper by Dr. R. F. Weir "On some new Methods of treating Hydrocele," in which he gives results of treatment by, and his preference for an operation practiced by Dr. R. J. Levis, of Philadelphia, of evacuating the sac by trocar, and injecting from one half to one drachm of the pure carbolic acid and allowing it to remain in the sac cavity. The pain produced by the acid is sharp, but momentary, as this agent is well known to produce a benumbing effect upon tissues to which it is applied.

The reaction from this operation is so slight, that generally the patient does not go to bed; but of necessity, the introduction of the trocar produces a considerable degree of pain, and the acid some too—hence it is not a *painless* operation.

Now there are certain cases of encysted hydrocele of the testis and the cord in which the evacuation by trocar would be either inapplicable or dangerous, because of liability to wound contained organs; or the sac being penetrated, owing to the thinness of its walls, they are liable to collapse upon withdrawal of its contents, and the injected fluid would be thrown into the surrounding tissues, and not in the sac, thus causing an inflammation that is always troublesome and not always devoid of danger.

In those cases where such methods can not be used the seton has been hitherto the only resource. This is not cleanly, is painful and irritating, and at times causes severe inflammation.

I have thus briefly outlined those methods of treatment most frequently used, shown their objections and the limit of their application, that the mode of operating suggested in this paper may be examined by contrast and properly estimated.

The operation is not original with me. About two years ago, in a short paragraph in some journal that I do not now remember, I read it. The writer had used it for a number of years, and during the war had operated on many cases in this way; and in no instance was the reaction enough to necessitate relief from military duty, or to produce the least additional inconvenience; in all cases the cure had been complete. The material used for injection had been from 20 to 40 drops of tincture of iodine.

Struck by the simplicity of the operation, its freedom from immediate or subsequent pain, and its efficiency in his cases, I was led to try it in two cases—all that I have treated since reading the article.

My first case was an old man upon whom I had previously operated by the old method of evacuation and iodine tincture injections, and without success. With a hypodermic syringe I inserted 30 drops of tincture of iodine. Within two days the reaction was sufficient to confine the patient to his room for a few days. A perfect cure resulted.

The other case was a young man with a rather recent and not very large hydrocele, in which I injected 20 drops of pure carbolic acid. This was followed by a scarcely perceptible increase of fluid, but had no pain and did not cause him to cease business—that of a salesman. The cure remains perfect up to present time.

In the case of the old gentleman the reaction was, perhaps, more than if I had used carbolic acid, as Dr. Levis' cases go to show its harmlessness in producing inflammation ; so, in future, I shall use only this remedy.

Now, what are the advantages of this operation over that of Dr. Levis'? Simply that it is painless in its performance, devoid of danger of throwing injection into surrounding tissues, less danger of wounding contained parts by the introduction of the instrument, equal curative efficiency, and a much wider application.

Its application can be universal, for it is just as easy to insert the point of a hypodermic needle into the sac of an encysted testicular, and encysted or diffuse hydrocele of cord, as it is to introduce a seton—the plan adopted in these varieties of hydrocele.

My cases, as far as they go, show it to be an efficient operation ; those of the author of the treatment show the same. Its adaptation to ends being established, its goodness as a surgical conduct follows ; its simplicity is attractive, and I hope those who have a wider field for surgical observation than I, will test the merits of the procedure and report results.

